# Supplementary material for: Identification of the Myogenetic Oligodeoxynucleotides (myoDNs) That Promote Differentiation of Skeletal Muscle Myoblasts by Targeting Nucleolin
Source: Front Cell Dev Biol. 2021 Jan 11;8:616706. doi: 10.3389/fcell.2020.616706 (PMC7874222; doi:10.3389/fcell.2020.616706)

Supplementary Material

Supplementary Data. The 899 iSN04-dependent DEGs defined as FPKM > 1.5-fold and FDR $p < 0.05$ between control and iSN04 groups.

Supplementary Movie. Simulated conformations of iSN04 at 310 K. 1737 conformations of iSN04 at 310 K were sequentially arrayed to make the movie. These conformations were taken from production run (see Materials and Methods in main text) by metropolis method. In the movie, hydrogen atoms were hidden to see the conformations clearly. The colors indicate the residues from 5' end in blue to 3' end in red as rainbow manner.

Supplementary Table S1. Sequences of PS-ODNs.

Name	Sequence (5'-3')	Reference
iSN01	AAAAGATTAGGGTGAGGG	Nigar et al., 2017
iSN02	AAAGATTAGGGTGAGGGT	Nigar et al., 2017
iSN03	AAGATTAGGGTGAGGGTG	Nigar et al., 2017
iSN04	AGATTAGGGTGAGGGTGA	Nigar et al., 2017
iSN04'	AAGTTAGGGTGAGGGTGA	This study
iSN05	GATTAGGGTGAGGGTGAG	Nigar et al., 2017
iSN06	ATTAGGGTGAGGGTGAGT	Nigar et al., 2017
iSN07	TTAGGGTGAGGGTGAGTT	Nigar et al., 2017
iSN08	AGTTCAACATTAGGGTGA	Nigar et al., 2017
iSN09	GTTCAACATTAGGGTGAA	Nigar et al., 2017
iSN10	TTCAACATTAGGGTGAAA	Nigar et al., 2017
iSN11	TCAACATTAGGGTGAAAA	Nigar et al., 2017
iSN12	CAACATTAGGGTGAAAAT	Nigar et al., 2017
iSN13	AACATTAGGGTGAAAATG	Nigar et al., 2017
iSN14	ACATTAGGGTGAAAATGA	Nigar et al., 2017
iSN15	CATTAGGGTGAAAATGAA	Nigar et al., 2017
iSN16	TAAAGCATTAGGGTGATG	Nigar et al., 2017
iSN17	AAAGCATTAGGGTGATGA	Nigar et al., 2017
iSN18	AAGCATTAGGGTGATGAA	Nigar et al., 2017
iSN19	AGCATTAGGGTGATGAAA	Nigar et al., 2017
iSN20	GCATTAGGGTGATGAAAT	Nigar et al., 2017
iSN21	CATTAGGGTGATGAAATC	Nigar et al., 2017
iSN22	ATTAGGGTGATGAAATCC	Nigar et al., 2017
iSN23	TTAGGGTGATGAAATCCA	Nigar et al., 2017
iSN24	ATCAGGCTCAAGCTTGAG	Nigar et al., 2017
iSN25	TCAGGCTCAAGCTTGAGT	Nigar et al., 2017
iSN26	CAGGCTCAAGCTTGAGTT	Nigar et al., 2017
iSN27	AGGCTCAAGCTTGAGTTC	Nigar et al., 2017
iSN28	GGCTCAAGCTTGAGTTCT	Nigar et al., 2017
iSN29	GCTCAAGCTTGAGTTGTG	Nigar et al., 2017
iSN30	CTCAAGCTTGAGTTCTGA	Nigar et al., 2017
iSN31	TCATTCCTAAGCTTGAGG	Nigar et al., 2017
iSN32	CATTCCTAAGCTTGAGGC	Nigar et al., 2017

iSN33	ATTCCTAAGCTTGAGGCC	Nigar et al., 2017
iSN34	TTCCTAAGCTTGAGGCCT	Nigar et al., 2017
iSN35	TCCTAAGCTTGAGGCCTA	Nigar et al., 2017
iSN36	CCTAAGCTTGAGGCCTAT	Nigar et al., 2017
iSN37	CTAAGCTTGAGGCCTATG	Nigar et al., 2017
iSN38	TAAGCTTGAGGCCTATGG	Nigar et al., 2017
iSN39	AAGCTTGAGGCCTATGGG	Nigar et al., 2017
iSN40	GGAACGATCCTCAAGCTT	Nigar et al., 2017
iSN41	GAACGATCCTCAAGCTTA	Nigar et al., 2017
iSN42	AACGATCCTCAAGCTTAG	Nigar et al., 2017
iSN43	ACGATCCTCAAGCTTAGG	Nigar et al., 2017
iSN44	CGATCCTCAAGCTTAGGT	Nigar et al., 2017
iSN45	GATCCTCAAGCTTAGGTC	Nigar et al., 2017
iSN46	TCCTCAAGCTTAGGTCCG	Nigar et al., 2017
iSN47	CCTCAAGCTTAGGTCCGC	Nigar et al., 2017
iSN48	AAATAGCTTTAGGGTTAG	Nigar et al., 2017
iSN49	AATAGCTTTAGGGTTAGC	Nigar et al., 2017
iSN50	ATAGCTTTAGGGTTAGCC	Nigar et al., 2017
iSN04$^{\Delta 15}$	AGATTAGGGTGAGGTGA	This study
iSN04$^{\Delta 14\text{-}15}$	AGATTAGGGTGAGTGA	This study
iSN04$^{\Delta 13\text{-}15}$	AGATTAGGGTGATGA	This study
iSN04^{T5G}	AGATGAGGGTGAGGGTGA	This study
iSN04^{G11T}	AGATTAGGGTTAGGGTGA	This study
iSN04$^{T5G/G11T}$	AGATGAGGGTTAGGGTGA	This study
CpG-2006	TCGTCGTTTTGTCGTTTTGTCGTT	Pohar et al., 2015
Tel-ODN	TTAGGGTTAGGGTTAGGGTTAGGG	Sackesen et al., 2013
AS1411†	GGTGGTGGTGGTTGTGGTGGTGGTGG	Girvan et al., 2006

† AS1411 has a phosphodiester backbone. Other ODNs were PS-ODNs.

Supplementary Table S2. Primer sequences for qPCR.

Gene	Sequence (5'-3')	Size (bp)	Reference
MYF5	AGAACTACTATAGCCTGCCGG<br>ATCTGTGGCATATACATTTGATACATCA	164	Zibat et al., 2010
MYH3	GGACAGGAAGAATGTGCTGAGATT<br>GCCTCTTGTAGGACTTGACTTTCAC	74	Norman et al., 2009
MYOD1	TGCTCCGACGGCATGATGGAC<br>TCGACACCGCCGCACTCT	127	Sharma et al., 2013
MYOG	AACCCAGGGGATCATCTGCTCAC<br>GTTGGGCATGGTTTCATCTGGGAAG	129	Sharma et al., 2013
NCL	ATTGGTAGCAACTCCTGGTAAG<br>CACTGTCATCATCCTCCTCTTC	110	Cai et al., 2018
PAX7	GACCCCTGCCTAACCACATC<br>GTCTCCTGGTAGCGGCAAAG	133	Sakai et al., 2017
TLR1	CTATACACCAAGTTGTCAGC<br>GTCTCCAACTCAGTAAGGTG	220	Yao et al., 2015
TLR2	GGCCAGCAAATTACCTGTGTG<br>AGGCGGACATCCTGAACCT	67	Hayashi et al., 2003
TLR3	TCCCAAGCCTTCAACGACTG<br>TGGTGAAGGAGAGCTATCCACA	68	Hayashi et al., 2003
TLR4	CTGCAATGGATCAAGGACCA<br>TTATCTGAAGGTGTTGCACATTCC	74	Hayashi et al., 2003
TLR5	TCGAGCCCCTACAAGGGAA<br>CACTGAGACTCTGCTATACAAGCTA	74	Hayashi et al., 2003
TLR6	CTATTGTTAAAAGCTTCCATTTTGT<br>ACCTGAAGCTCAGCGATGTAGTTC	187	Hayashi et al., 2003
TLR7	TTACCTGGATGGAAACCAGCTAC<br>TCAAGGCTGAGAAGCTGTAAGCTA	72	Hayashi et al., 2003
TLR8	GAGAGCCGAGACAAAAACGTTC<br>TGTCGATGATGGCCAATCC	73	Hayashi et al., 2003
TLR9	TGGTGTTGAAGGACAGTTCTCTC<br>CACTCGGAGGTTTCCCAGC	71	Hayashi et al., 2003
TLR10	GAAAGGTTCCCGCAGACTTG<br>TGGAGTTGAAAAAGGAGGTTATAG	73	Hayashi et al., 2003

YWHAZ	CAAGCATACCAAGAAGCATTTGA<br>GGGCCAGACCCAGTCTGA	76	Nihashi et al., 2019
Tlr1	TCTCTGAAGGCTTTGTCGATACA<br>GACAGAGCCTGTAAGCATATTCG	212	Caramalho et al., 2003
Tlr2	TCTAAAGTCGATCCGCGACAT<br>TACCCAGCTCGCTCACTACGT	344	Caramalho et al., 2003
Tlr3	TTGTCTTCTGCACGAACCTG<br>CGCAACGCAAGGATTTTATT	205	Caramalho et al., 2003
Tlr4	CAAGAACATAGATCTGAGCTTCAACCC<br>GCTGTCCAATAGGGAAGCTTTCTAGAG	278	Caramalho et al., 2003
Tlr5	ACTGAATTCCTTAAGCGACGTA<br>AGAAGATAAAGCCGTGCGAAA	401	Caramalho et al., 2003
Tlr6	AACAGGATACGGAGCCTTGA<br>CCAGGAAAGTCAGCTTCGTC	199	Caramalho et al., 2003
Tlr7	TTCCGATACGATGAATATGCACG<br>TGAGTTTGTCCAGAAGCCGTAAT	412	Caramalho et al., 2003
Tlr8	GGCACAACTCCCTTGTGATT<br>CATTTGGGTGCTGTTGTTTG	195	Caramalho et al., 2003
Tlr9	TGCAATTGGCTGTTCCTGAA<br>GGTGGTGGATACGGTTGGAG	100	Saluja et al., 2012
Tlr11	CCAGGACTGCACCTTTTGG<br>GTGACACTGGTTGTACGCAAT	185	This study
Tlr12	AGAGCTGGCTGGTATGTTCC<br>GTGTTCTTGTCAGGTCCAGAATC	161	This study
Tlr13	GGAGCGCCTTGATCTAACTAACA<br>TCAGGTGGGTCAGAGAAACCA	80	This study

Supplementary Table S3. The number of the reads obtained by RNA-seq.

Sample	Raw reads	Cleaned reads	Mapped reads	% mapped
Control-A	59,865,454	58,169,640	53,874,761	92.3%
Control-B	51,357,760	50,323,502	46,717,465	92.8%
Control-C	46,217,046	42,965,166	39,340,050	91.6%
iSN04-A	47,556,514	46,737,876	43,434,950	92.9%
iSN04-B	45,959,342	45,008,402	41,670,855	92.6%
iSN04-C	56,638,202	54,726,942	50,462,497	92.2%

Supplementary Table S4. Ionic components of Ham's F10 medium.

Components	Concentration
$CaCl_2$	0.3 mM
$CuSO_4$	1.0×10^{-5} mM
$FeSO_4$	1.0×10^{-3} mM
$MgSO_4$	0.6 mM
KCl	3.8 mM
KH_2PO_4	0.6 mM
$NaHCO_3$	14.3 mM
NaCl	127.6 mM
Na_2HPO_4	1.1 mM
$ZnSO_4$	1.0×10^{-4} mM

Supplementary Table S5. Summary of mass spectrometry.

	iSN04-5'-bio	iSN04-3'-bio
No. of proteins identified	14	74
No. of unique peptides	98	521
No. of spectra matching	116	715

Supplementary Table S6. Results of mass spectrometry for iSN04-binding protein.

Protein	Accession No.	Molecular weight	iSN04-5'-bio	iSN04-3'-bio
Nucleolin	P09405, NUCL_MOUSE	77 kDa	42	132
Heat shock protein HSP 90-beta	P11499, HS90B_MOUSE	83 kDa	4	41
DNA topoisomerase 1	Q04750, TOP1_MOUSE	91 kDa	10	37
Heat shock protein HSP 90-alpha	P07901, HS90A_MOUSE	85 kDa	0	36
Eukaryotic translation initiation factor 4 gamma 2	Q62448, IF4G2_MOUSE	102 kDa	0	20
Elongation factor 2	P58252, EF2_MOUSE	95 kDa	0	18
Junctophilin-2	Q9ET78, JPH2_MOUSE	75 kDa	0	18
Nucleolar transcription factor 1	P25976, UBF1_MOUSE	90 kDa	0	13
40S ribosomal protein S3	P62908, RS3_MOUSE	27 kDa	0	10
Transitional endoplasmic reticulum ATPase	Q01853, TERA_MOUSE	89 kDa	0	10
Cyclin-dependent kinase 11B	P24788, CD11B_MOUSE	92 kDa	0	8
Aspartyl/asparaginyl beta-hydroxylase	Q8BSY0, ASPH_MOUSE	83 kDa	0	7
Splicing factor, proline- and glutamine-rich	Q8VIJ6, SFPQ_MOUSE	75 kDa	2	7
Plasminogen activator inhibitor 1 RNA-binding protein	Q9CY58, PAIRB_MOUSE	45 kDa	2	7
AP-2 complex subunit beta	Q9DBG3, AP2B1_MOUSE	105 kDa	0	7
AP-2 complex subunit alpha-2	P17427, AP2A2_MOUSE	104 kDa	0	6
40S ribosomal protein S2	P25444, RS2_MOUSE	31 kDa	0	6
60S ribosomal protein L8	P62918, RL8_MOUSE	28 kDa	0	6
Epidermal growth factor receptor kinase substrate 8	Q08509, EPS8_MOUSE	92 kDa	0	6

tRNA (cytosine(34)-C(5))-methyltransferase	Q1HFZ0, NSUN2_MOUSE	85 kDa	0	6
Eukaryotic translation initiation factor 3 subunit B	Q8JZQ9, EIF3B_MOUSE	91 kDa	0	6
40S ribosomal protein S9	Q6ZWN5, RS9_MOUSE	23 kDa	0	5
Mannosyl-oligosaccharide glucosidase	Q80UM7, MOGS_MOUSE	92 kDa	0	5
Desmoplakin	E9Q557, DESP_MOUSE	333 kDa	0	4
Elongation factor 1-alpha 1	P10126, EF1A1_MOUSE	50 kDa	0	4
Putative ATP-dependent RNA helicase Pl10	P16381, DDX3L_MOUSE	73 kDa	0	4
ADP/ATP translocase 1	P48962, ADT1_MOUSE	33 kDa	0	4
DNA replication licensing factor MCM5	P49718, MCM5_MOUSE	82 kDa	0	4
Actin, cytoplasmic 1	P60710, ACTB_MOUSE	42 kDa	0	4
40S ribosomal protein S6	P62754, RS6_MOUSE	29 kDa	0	4
Nuclear receptor subfamily 4 group A member 2	Q06219, NR4A2_MOUSE	67 kDa	5	4
Probable ATP-dependent RNA helicase DDX17	Q501J6, DDX17_MOUSE	72 kDa	0	4
Eukaryotic translation initiation factor 4B	Q8BGD9, IF4B_MOUSE	69 kDa	0	4
26S proteasome non-ATPase regulatory subunit 2	Q8VDM4, PSMD2_MOUSE	100 kDa	0	4
ATP-dependent RNA helicase DDX1	Q91VR5, DDX1_MOUSE	83 kDa	0	4
Polypyrimidine tract-binding protein 1	P17225, PTBP1_MOUSE	56 kDa	0	3
60S ribosomal protein L3	P27659, RL3_MOUSE	46 kDa	0	3
60S ribosomal protein L13	P47963, RL13_MOUSE	24 kDa	0	3
60S ribosomal protein L9	P51410, RL9_MOUSE	22 kDa	0	3
40S ribosomal protein S25	P62852, RS25_MOUSE	14 kDa	0	3
60S ribosomal protein L10-like	P86048, RL10L_MOUSE	25 kDa	0	3

40S ribosomal protein S3a	P97351, RS3A_MOUSE	30 kDa	0	3
Lymphocyte-specific helicase	Q60848, HELLS_MOUSE	95 kDa	0	3
Cytoskeleton-associated protein 4	Q8BMK4, CKAP4_MOUSE	64 kDa	0	3
Elongin-A	Q8CB77, ELOA1_MOUSE	87 kDa	0	3
Ras and Rab interactor 1	Q921Q7, RIN1_MOUSE	83 kDa	0	3
Inversin	O89019, INVS_MOUSE	117 kDa	0	2
Vitronectin	P29788, VTNC_MOUSE	55 kDa	0	2
40S ribosomal protein S20	P60867, RS20_MOUSE	13 kDa	0	2
Heterogeneous nuclear ribonucleoprotein K	P61979, HNRPK_MOUSE	51 kDa	0	2
40S ribosomal protein S14	P62264, RS14_MOUSE	16 kDa	0	2
40S ribosomal protein S11	P62281, RS11_MOUSE	18 kDa	0	2
40S ribosomal protein S4, X isoform	P62702, RS4X_MOUSE	30 kDa	0	2
60S ribosomal protein L22	P67984, RL22_MOUSE	15 kDa	0	2
40S ribosomal protein S5	P97461, RS5_MOUSE	23 kDa	0	2
Mitogen-activated protein kinase kinase kinase kinase 5	Q8BPM2, M4K5_MOUSE	95 kDa	0	2
La-related protein 4	Q8BWW4, LARP4_MOUSE	80 kDa	0	2
NFX1-type zinc finger-containing protein 1	Q8R151, ZNFX1_MOUSE	219 kDa	0	2
Activating signal cointegrator 1 complex subunit 2	Q91WR3, ASCC2_MOUSE	86 kDa	0	2
Probable ATP-dependent RNA helicase DDX27	Q921N6, DDX27_MOUSE	86 kDa	0	2
Mitogen-activated protein kinase kinase kinase kinase 3	Q99JP0, M4K3_MOUSE	101 kDa	0	2
60S ribosomal protein L15	Q9CZM2, RL15_MOUSE	24 kDa	0	2
Elongation factor 1-gamma	Q9D8N0, EF1G_MOUSE	50 kDa	0	2

N-terminal kinase-like protein	Q9EQC5, SCYL1_MOUSE	89 kDa	0	2
Ribosomal protein S6 kinase alpha-4	Q9Z2B9, KS6A4_MOUSE	86 kDa	0	2
Stress-70 protein, mitochondrial	P38647, GRP75_MOUSE	73 kDa	2	0
Adenosylhomocysteinase	P50247, SAHH_MOUSE	48 kDa	3	0
ATP synthase subunit alpha, mitochondrial	Q03265, ATPA_MOUSE	60 kDa	2	0

Numerals are the number of the identified proteins in the samples.

Supplementary Figure Legends

Supplementary Figure S1. Screening of the PS-ODNs promoting myoblast differentiation. Representative immunofluorescent images of the mMBs treated with 10 µM PS-ODNs in GM for 48 h (screening system).

Supplementary Figure S2. Identification of myoDNs. (**A**) Ratio of MHC^+ cells within the another lot of mMBs treated with 10 µM PS-ODNs in GM for 48 h (screening system). ** $p < 0.01$ vs control (Dunnett's test). $n = 3$. (**B**) Relative number of the $DAPI^+$ cells of the mMBs shown in panel A. The mean value of the control samples was set at 1.0. ** $p < 0.01$ vs control (Dunnett's test). $n = 3$. (**C**) Ratio of MHC^+ cells within the mMBs treated with 10 µM of iSN04' or iSN04 in GM for 48 h (screening system). ** $p < 0.01$ vs control; NS, no significant difference (Scheffe's *F* test). $n = 3$. (**D**) Representative immunofluorescent images of the C2C12 cells treated with 10 µM iSN04 in DM for 48 h. Scale bar, 200 µm. Ratio of MHC^+ cells and multinuclear myotubes were quantified. * $p < 0.05$, ** $p < 0.01$ vs control (Student's *t* test). $n = 8$. (**E**) Sequences of myoDNs, iSN01-07. Telomeric sequences are underlined. (**F**) Ratio of MHC^+ cells within the mMBs treated with 10 µM of iSN02 or denatured-iSN02 (de-iSN02) in GM for 48 h (screening system). PS-ODNs were denatured at 95°C for 5 min then immediately placed on ice. ** $p < 0.01$ vs control, †† $p < 0.01$ vs iSN02 (Scheffe's *F* test). $n = 3$. (**G**) Ratio of MHC^+ cells within the mMBs treated with 10 µM of iSN04 or

denatured-iSN04 (de-iSN04) in GM for 48 h (screening system). ** $p < 0.01$ vs control; NS, no significant difference (Scheffe's F test). $n = 3$.

Supplementary Figure S3. iSN04 activity is independent of TLR signal. (**A**) RT-PCR (40 cycles) result of human TLR gene expression in the hMBs maintained in GM. (**B**) RT-PCR (40 cycles) results of murine TLR gene expression in the mMBs and C2C12 cells maintained in GM. (**C**) Heatmap of FPKM values of the genes involved in TLR signaling pathway (KEGG pathway; map04620) in the hMBs treated with 30 μM iSN04 for 24 h. There was no significant difference between control and the iSN04-treated groups in each gene expression (FDR).

Supplementary Figure S4. Profile of iSN04-dependent gene expression. (**A**) Qualities of the RNA samples used for RNA-seq. The RIN values measured by Agilent 2100 Bioanalyzer are 10.0 (max score) in all samples. (**B**) FPKM values of myogenic gene transcripts in the hMBs treated with 30 μM iSN04 for 24 h. * $p < 0.05$, ** $p < 0.01$ vs control (FDR). $n = 3$.

Supplementary Figure S5. Berberine analogs enhance iSN04 activity. (**A**) Structural formula of berberine analogs. (**B**) Representative images of agarose gel electrophoresis of iSN04 mixed with berberine in cationic solutions. The gels were scanned as described in Figure 3E. (**C**) Representative images of agarose gel electrophoresis of iSN01-iSN07 mixed

with berberine in F10 medium. (**D**) Representative images of agarose gel electrophoresis of iSN04 and AS1411 mixed with berberine in F10 medium.

Supplementary Figure S6. iSN04 targets nucleolin and improves p53 protein level. (**A**) Representative immunofluorescent images of the C2C12 cells maintained in DM at day 0 and 2. Scale bar, 50 µm. (**B**) Representative immunofluorescent images of the C2C12 cells treated with 3 µM of iSN04 or AS1411 in DM for 48 h. Scale bar, 50 µm. (**C**) qPCR results of nucleolin gene expression in the hMBs maintained in DM. Mean value of hMBs at day 0 was set to 1.0. ** $p < 0.01$ vs day 0 (Scheffe's F test). $n = 3$. (**D**) FPKM values of nucleolin transcripts in the hMBs treated with 30 µM iSN04 in DM for 24 h. $n = 3$. (**E**) Heatmap of FPKM values of the iSN04-dependent DEGs enriched in the p53 signaling pathway (KEGG pathway; hsa04115) shown in Figure 4E. (**F**) FPKM values of p53 transcripts in the hMBs treated with 30 µM iSN04 in DM for 24 h. ** $p < 0.01$ vs control (FDR). $n = 3$. (**G**) The structure of AS1411 determined by solution NMR (Protein Data Bank, 2N3M). (**H**) Representative immunofluorescent image of MEFs. Scale bar, 50 µm. (**I**) Western blotting (5 µg protein/lane) of nucleolin and GAPDH in the soluble whole cell lysates of C2C12 and MC3T3-E1 cells used for precipitation assay.

Supplementary References

Cai, X. W., Yu, W. W., Yu, W., Zhang, Q., Feng, W., Liu, M. N., et al. (2018). Tissue-based quantitative proteomics to screen and identify the potential biomarkers for early recurrence/metastasis of esophageal squamous cell carcinoma. *Cancer Med.* 7, 2504-2517. doi: 10.1002/cam4.1463

Girvan, A. C., Teng, Y., Casson, L. K., Thomas, S. D., Juliger, S., Ball, M. W., et al. (2006). AGRO100 inhibits activation of nuclear factor-kappaB (NF-kappaB) by forming a complex with NF-kappaB essential modulator (NEMO) and nucleolin. *Mol. Cancer Ther* 5, 1790-1799. doi: 10.1158/1535-7163.MCT-05-0361

Hayashi, F., Means, T. K., and Luster, A. D. (2003). Toll-like receptors stimulate human neutrophil function. *Blood* 102, 2660-2609. doi: 10.1182/blood-2003-04-1078

Nigar, S., Yamamoto, Y., Okajima, T., Shigemori, S., Sato, T., Ogita, T., et al. (2017). Synergistic oligodeoxynucleotide strongly promotes CpG-induced interleukin-6 production. *BMC Immunol.* 18, 44. doi: 10.1186/s12865-017-0227-7

Nihashi, Y., Umezawa, K., Shinji, S., Hamaguchi, Y., Kobayashi, H., Kono, T., et al. (2019). Distinct cell proliferation, myogenic differentiation, and gene expression in skeletal muscle myoblasts of layer and broiler chickens. *Sci. Rep.* 9, 16527. doi: 10.1038/s41598-019-52946-4

Norman, H., Zackrisson, H., Hedstrom, Y., Andersson, P., Nordquist, J., Eriksson, L. I., et al. (2009). Myofibrillar protein and gene expression in

acute quadriplegic myopathy. *J. Neurol. Sci.* 285, 28-38. doi: 10.1016/j.jns.2009.04.041

Pohar, J., Lainscek, D., Fukui, R., Yamamoto, C., Miyake, K., Jerala, R., et al. (2015). Species-specific minimal sequence motif for oligodeoxyribonucleotides activating mouse TLR9. *J. Immunol.* 195, 4396-4405. doi: 10.4049/jimmunol.1500600

Sackesen, C., van de Veen, W., Akdis, M., Soyer, O., Zumkehr, J., Ruckert, B., et al. (2013). Suppression of B-cell activation and IgE, IgA, IgG1 and IgG4 production by mammalian telomeric oligonucleotides. *Allergy* 68, 593-603. doi: 10.1111/all.12133

Sakai, H., Fukuda, S., Nakamura, M., Uezumi, A., Noguchi, Y. T., Sato, T., et al. (2017). Notch ligands regulate the muscle stem-like state ex vivo but are not sufficient for retaining regenerative capacity. *PLoS One* 12: e0177516. doi: 10.1371/journal.pone.0177516

Saluja, R., Delin, I., Nilsson, G. P., and Adner, M. (2012). FcεR1-mediated mast cell reactivity is amplified through prolonged Toll-like receptor-ligand treatment. *PLoS One* 7: e43547. doi: 10.1371/journal.pone.0043547

Sharma, V., Harafuji, N., Belayew, A., and Chen, Y. W. (2013). DUX4 differentially regulates transcriptomes of human rhabdomyosarcoma and mouse C2C12 cells. *PLoS One* 8: e64691. doi: 10.1371/journal.pone.0064691

Yao, C., Oh, J. H., Lee, D. H., Bae, J. S., Jin, C. L., Park, C. H., et al. (2015). Toll-like receptor family members in skin fibroblasts are functional and

have a higher expression compared to skin keratinocytes. *Int. J. Mol. Med.* 35, 1443-1450. doi: 10.3892/ijmm.2015.2146

Zibat, A., Missiaglia, E., Rosenberger, A., Pritchard-Jones, K., Shipley, J., Hahn, H., et al. 2010. Activation of the hedgehog pathway confers a poor prognosis in embryonal and fusion gene-negative alveolar rhabdomyosarcoma. *Oncogene* 29, 6323-6330. doi: 10.1038/onc.2010.368

Supplementary Figure S1

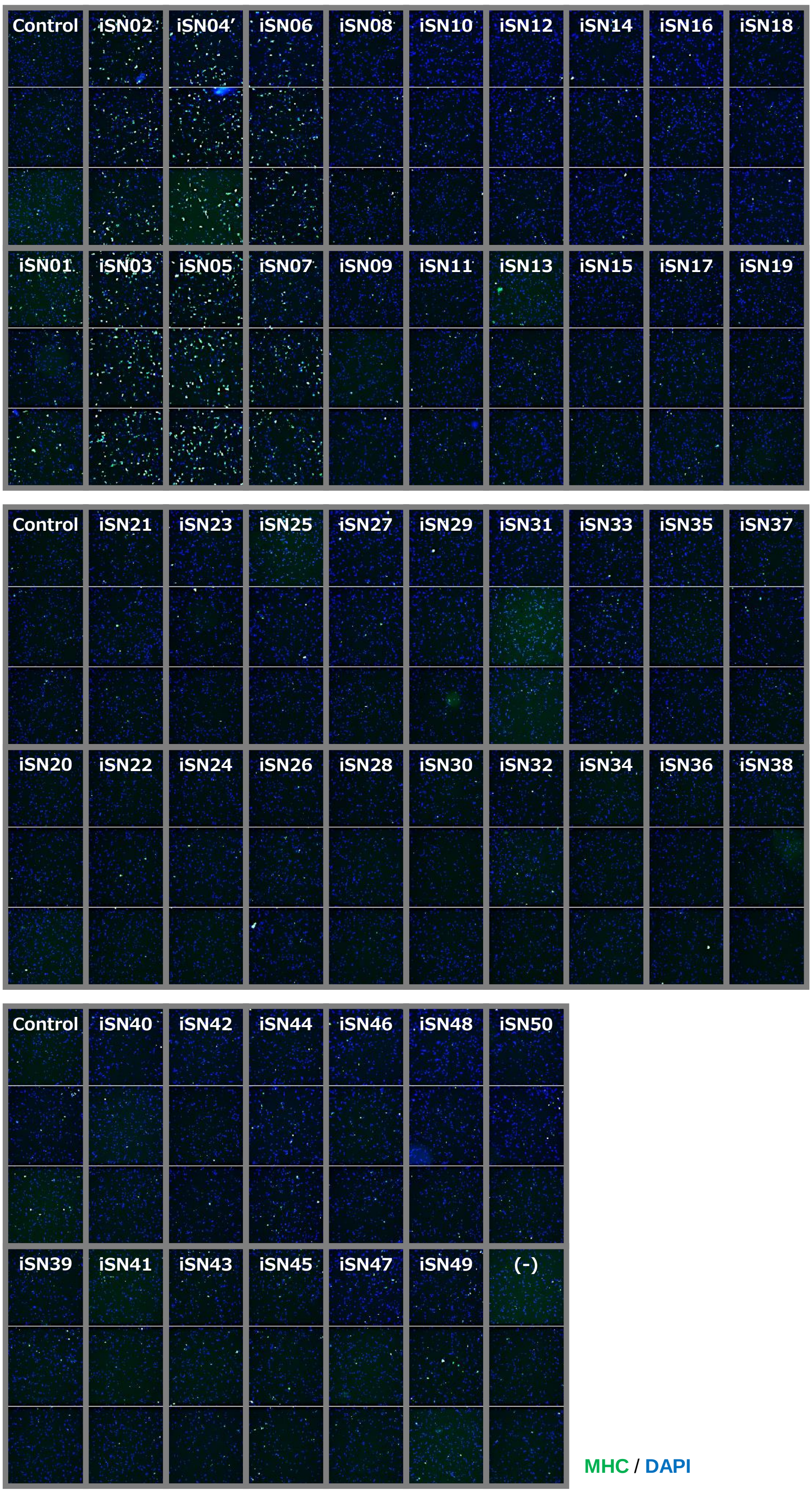

Supplementary Figure S2

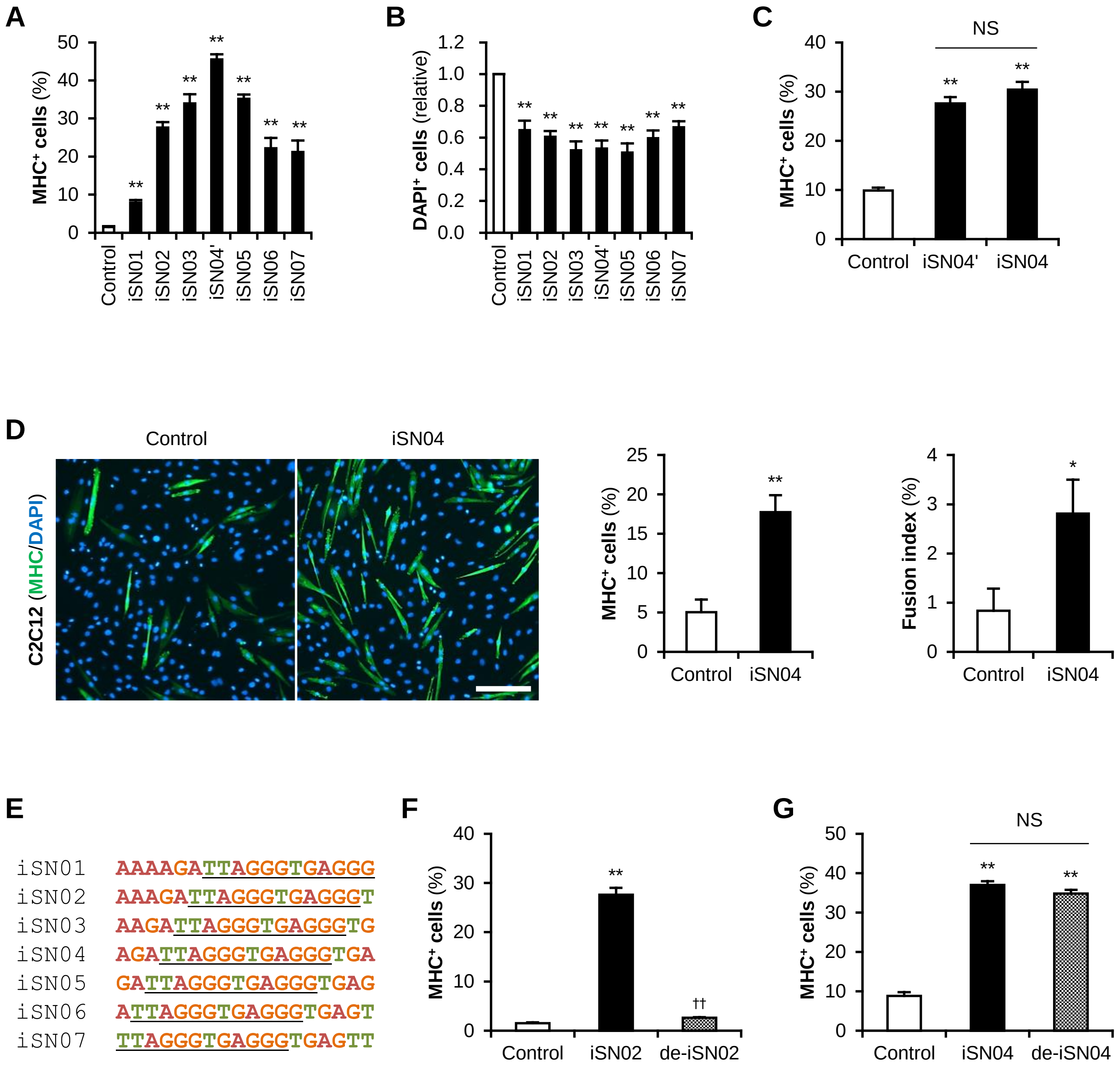

Supplementary Figure S3

A

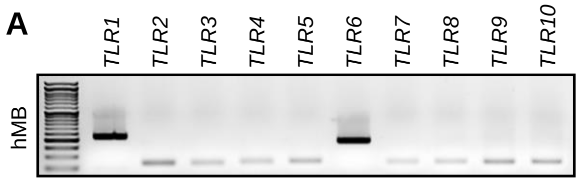

B

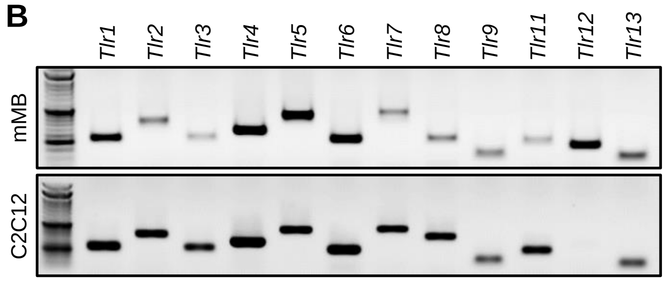

C

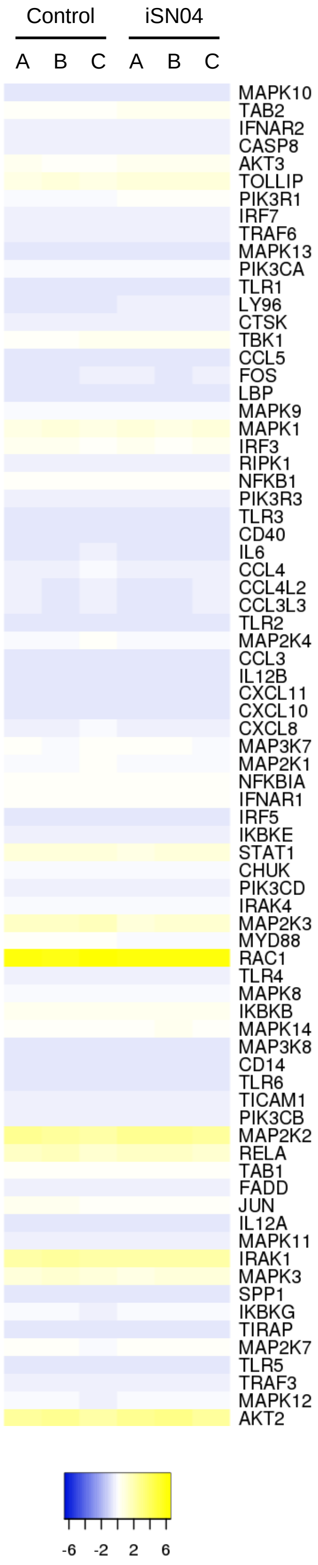

Supplementary Figure S4

A

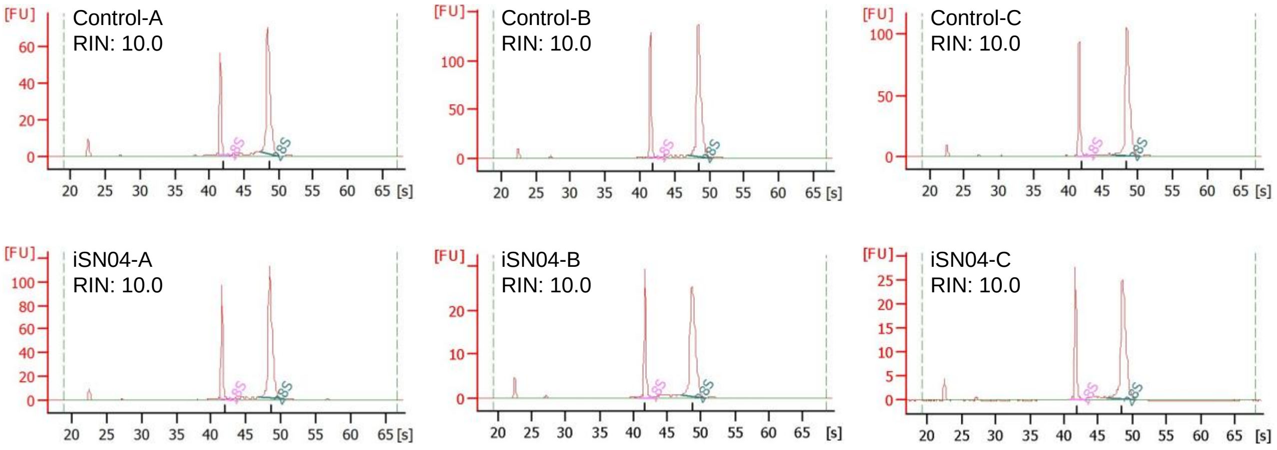

B

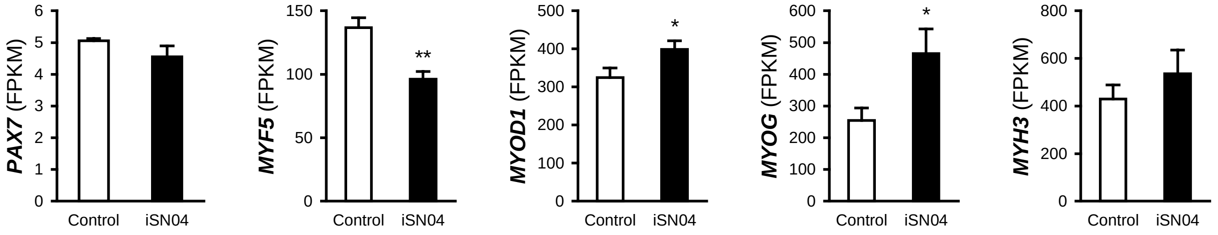

Supplementary Figure S5

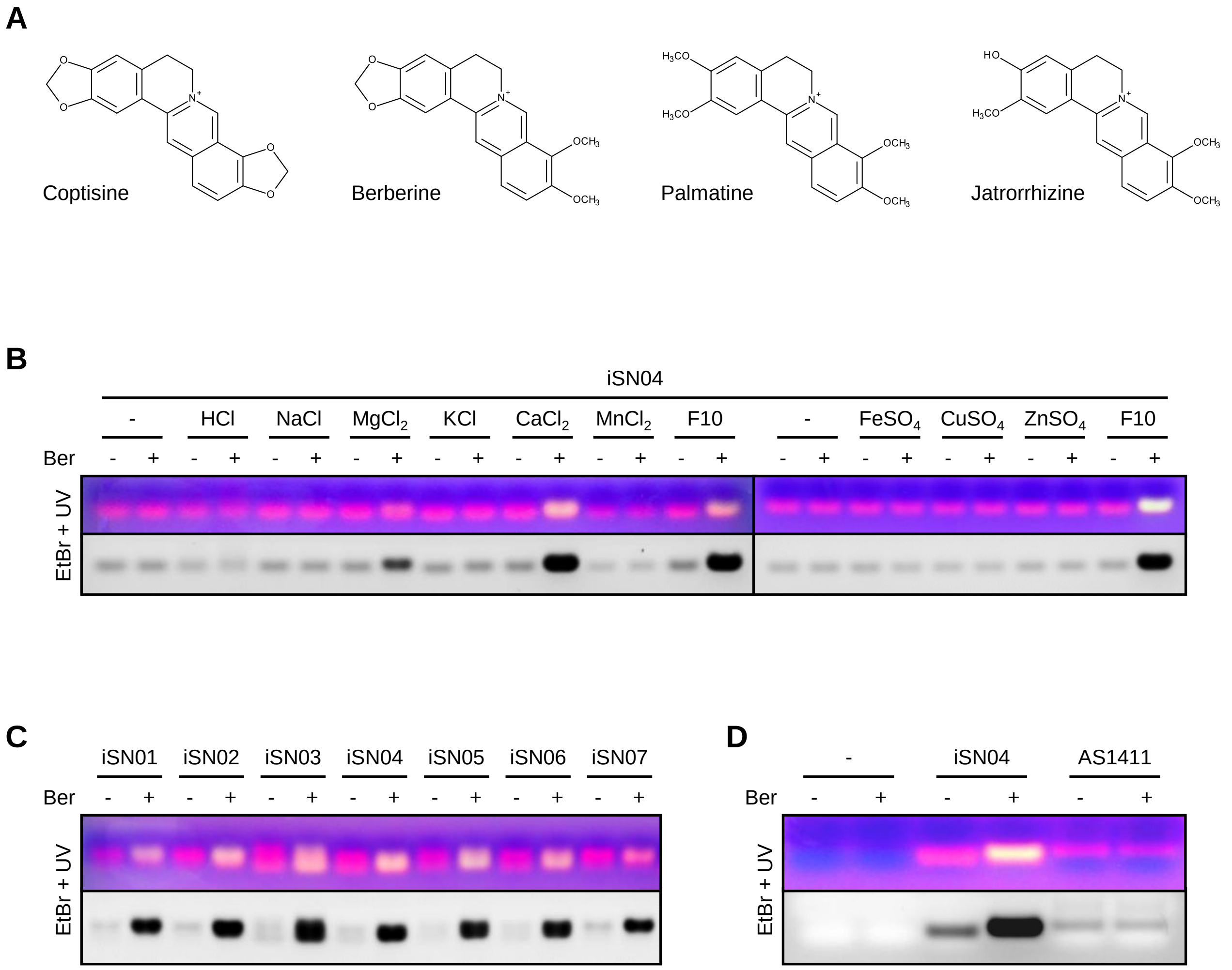

Supplementary Figure S6

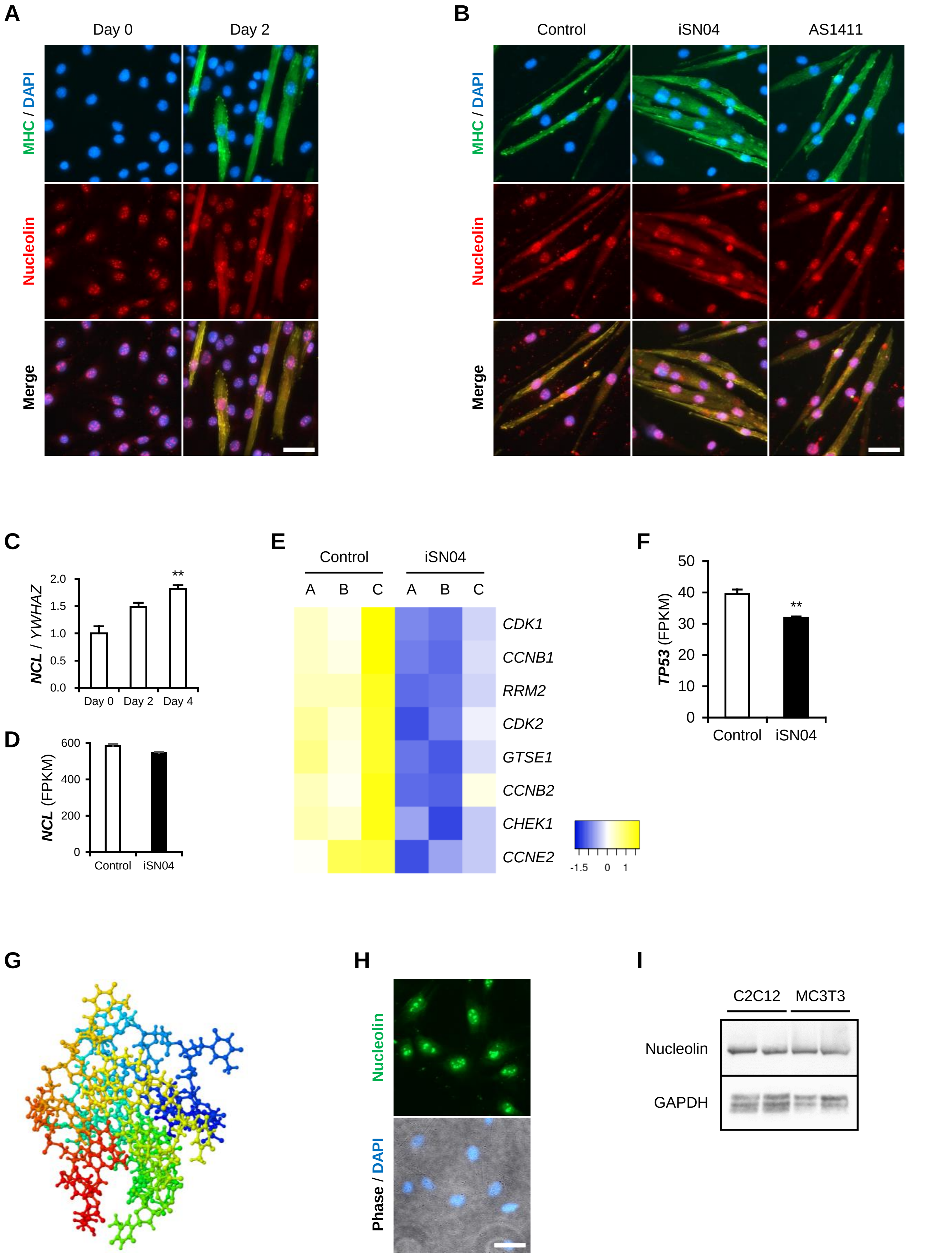

Supplement: Supplementary file 1 [file Data_Sheet_1.PDF]
